# Randomized Double-Blind Placebo-Controlled Supplementation with Standardized *Terminalia chebula* Fruit Extracts Reduces Facial Sebum Excretion, Erythema, and Wrinkle Severity

**DOI:** 10.3390/jcm12041591

**Published:** 2023-02-17

**Authors:** Mincy Chakkalakal, Adrianne Pan, Dawnica Nadora, Nimrit Gahoonia, Ratan K. Chaudhuri, Waqas Burney, Shivani Thacker, Anastasia Shakhbazova, Chaitra Subramanyam, Cindy J. Chambers, Raja K. Sivamani

**Affiliations:** 1 Integrative Skin Science and Research, Sacramento, CA 95815, USA; 2 College of Medicine, California Northstate University, Elk Grove, CA 95757, USA; 3 College of Osteopathic Medicine, Touro University California, Vallejo, CA 94592, USA; 4 Sytheon, 10 Waterview Blvd, Parsippany, NJ 07054, USA; 5 College of Osteopathic Medicine, Western University of Health Sciences, Pomona, CA 91766, USA; 6 School of Medicine, University of California, Riverside, CA 92521, USA; 7 Pacific Skin Institute, Sacramento, CA 95815, USA; 8 Department of Dermatology, University of California-Davis, Sacramento, CA 95816, USA

**Keywords:** *Terminalia chebula*, haritaki, herbs, sebum, wrinkles

## Abstract

*Terminalia chebula* (TC) is a medicinal plant that exhibits antioxidant, anti-inflammatory, and antibacterial properties and that is widely used in Ayurveda and herbal formulations. However, the skin effects of TC as an oral supplement have not been studied. The objective of this study is to determine if oral TC fruit extract supplementation can modulate the skin’s sebum production and reduce the appearance of wrinkles. A prospective double-blind placebo-controlled study was conducted on healthy females aged 25–65. Subjects were supplemented with an oral placebo or *Terminalia chebula* (250 mg capsule, Synastol TC) capsules twice daily for eight weeks. A facial image collection and analysis system was used to assess the facial appearance of wrinkle severity. Standardized, non-invasive tools were used to measure facial moisture, sebum production, transepidermal water loss, melanin index and erythema index. For those who had a baseline sebum excretion rate >80 ug/cm^2^, TC supplementation produced a significant decrease in forehead sebum excretion rate compared to the placebo at four weeks (−17 decrease vs. 20% increase, *p* = 0.07) and at eight weeks (−33% decrease vs. 29% increase, *p* < 0.01). Cheek erythema decreased by 2.2% at eight weeks, while the placebo treatment increased cheek erythema by 1.5% (*p* < 0.05). Facial wrinkles decreased by 4.3% in the TC group and increased by 3.9% in the placebo group after eight weeks of supplementation (*p* < 0.05). TC supplementation reduces facial sebum and improves the appearance of wrinkles. Future studies should consider evaluating oral TC as adjuvant therapy for acne vulgaris.

## 1. Introduction

*Terminalia chebula* Retz. (Combraetaceae), also known as chebulic myrobalan or haritaki, is a deciduous tree native to South Asia. The fruit of the tree is known for its widespread use in traditional medicine and is recognized as a source of therapeutic agents to treat diverse health conditions (including diabetes, atherosclerosis, constipation, hemorrhoids, arthritis, and allergies) and whose fruits have been reported to display anti-bacterial, anti-fungal, antioxidant and anti-inflammatory properties [1,2]. The medicinal properties of *Terminalia chebula* (TC) may be attributed to the diverse phytoconstituents that are found in the fruit. These include hydrolyzable tannins, flavonoids, anthraquinones, galloyl glucose, saponins, and sterols.

There has been growing interest in the use of TC for both the gut and the skin health. For example, non-standardized extracts of *Terminalia chebula* have demonstrated photoprotective properties [3,4]. Non-standardized TC has also been effectively formulated with retinol and niacinamide in topical lotions for anti-aging effects [5]. A recent study with 1% lotion of standardized TC fruit extract has shown longer-lasting and more efficient neutralization of reactive oxygen species (ROS) than natural tocopherol; treatment of keratinocytes with TC prior to being stressed with urban dust safeguarded against increases in intracellular ROS, inhibited release of inflammatory cytokines IL-6 and IL-8, and protected membrane lipids against peroxidation [3]. A clinical study further demonstrated statistically significant improvements in dermatologist scores and subject self-assessments for skin texture, hydration, tone, firmness, and radiance as compared to placebo [3].

There is growing interest in the role of the microbiome in relation to gut health. Improvement of the gut microbiome has been associated with impact on the local gut epithelial cells [6], the immune system [7], and chronic metabolic and skin diseases [8,9,10]. Support of the gut through supplementation has been associated with improvement of the gut microbial diversity and increase in the production of short chain fatty acids [11,12]. Modulation of the gut microbiome has been associated with changes in the skin, supporting the presence of a gut-skin axis [13,14].

Recent studies have also demonstrated the effects of TC on the gut. TC is part of the well-known Ayurvedic formulation Triphala that consists of *Emblica officianalis*, *Terminalia belerica*, and *Terminalia chebula* fruit extracts. Previous studies of Triphala have shown that it can diversify the gut microbiome [11]. Other studies that were specific to TC showed that it can inhibit pathogenic gut bacteria [15] and has intestinal and gastric prokinetic effects in mice and rats to support its use in constipation [16,17,18]. Furthermore, the oral ingestion of Triphala has been shown to reduce scalp sebum excretion rate [19,20]. Taken together, there is evidence that supports the notion that TC has an impact on the gut and the skin and may influence the gut-skin axis.

Tannins such as chebulagic acid and chebulinic acid, as well as other minor tannins may account for the majority of the skin benefits of TC extract [21,22,23,24], but the abundance, stability and bioactivity of these compounds can vary depending upon the extraction protocol used, for example, with respect to the tree component chosen as starting material, solvents used during extraction, and excipients used to generate a final topical product. These aspects of protocol design have not been consistently described in published literature, however, and some protocols may yield extracts as low as 20% of hydrolyzable tannins [25]. In the current study, we have used a standardized water-based extraction process that reliably contains 70% hydrolyzable tannins, with high contents of chebulinic acid (≥20%) and chebulagic acid (≥15%).

The objective of this study is to determine if oral TC supplementation with highly enriched hydrolyzable tannins (about 70%) can alter the skin’s properties. More specifically, we investigated whether oral TC supplementation would reduce skin sebum excretion rate and reduce the appearance of wrinkles. To the best of our knowledge, no prior oral clinical study has evaluated the effects of the oral intake of standardized or non-standardized TC extract on facial skin.

## 2. Material and Methods

### 2.1. Materials

**Figure 1:**
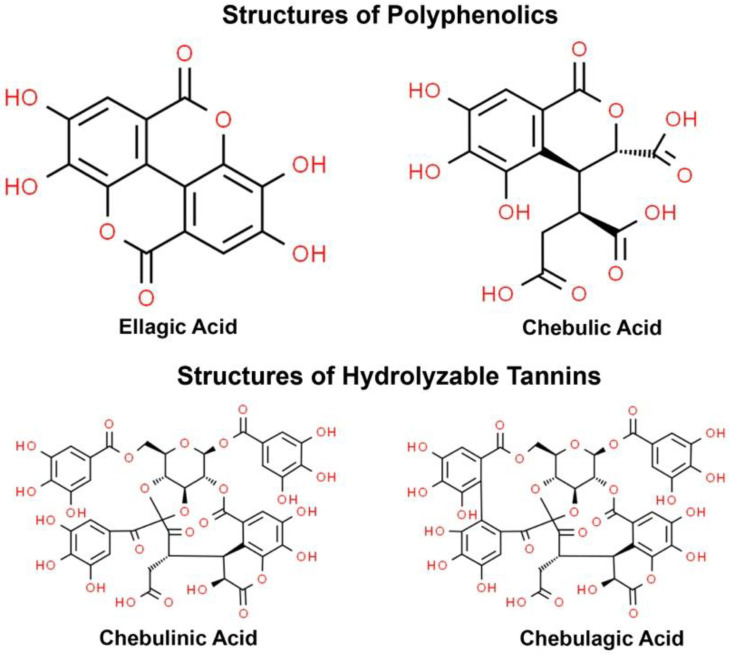
Chemicals structures of ellagic acid, chebulic acid, chebulinic acid, and chebulagic acid that are found in the standardized extract of *Terminalia chebula*.

The standardized TC fruit extract used in this study is a commercially available product from Sytheon (Parsippany, NJ, USA) called Synastol^®^ TC, which is standardized against hydrolysable tannins (70%) containing two key bioactive compounds, chebulinic acid (≥20%) and chebulagic acid (≥10%) and having a free gallic acid content of ≤5%. The chemicals structures are shown in Figure 1.

Interventional capsule (hard gelatin capsule shells, size 1, from Alfa Caps) composition consists of 250 mg TC (from Sytheon, Parsippany, NJ USA), Microcrystalline cellulose USP/NF (from UPI Chem, Mebane, NC USA), Starch NF (Maize, from Spectrum Chemicals, New Brunswick, NJ, USA), Cab-O-Sil (M-5F Untreated fumes silica; from Cabot Corporation, Alpharetta, GA, USA), and Stearic acid USP/NF (from UPI, Mebane, NC, USA). The Placebo did not contain any TC.

### 2.2. Study Design, Recruitment and Randomization

This double-blind placebo controlled clinical trial was conducted between October 2020 and March 2022. The study was approved by the Institutional Review Board (IntegReview Ltd.) and registered at www.clinicaltrials.gov (NCT04597502). All participants provided written informed consent prior to participation. All study visits were conducted in the greater Sacramento area at Integrative Skin Science and Research. Enrolled study subjects were randomized to 250 mg capsules twice daily for 8 weeks of TC or an oral placebo twice daily for 8 weeks. Block randomization was created a priori and blindly allocated through blinded sealed envelopes by the clinical coordinators.

Subjects attended a baseline, 4-week and 8-week visit at which several biophysical properties of the skin were measured such as facial sebum production and facial erythema. Facial photographs for image analysis were obtained at each visit. All measurements were performed after subjects rested for at least 15 min in a temperature (approximately 70 °F) and humidity-controlled room to acclimatize to the ambient conditions. All non-invasive measurements and photography were performed by trained clinical coordinators.

**Figure 2:**
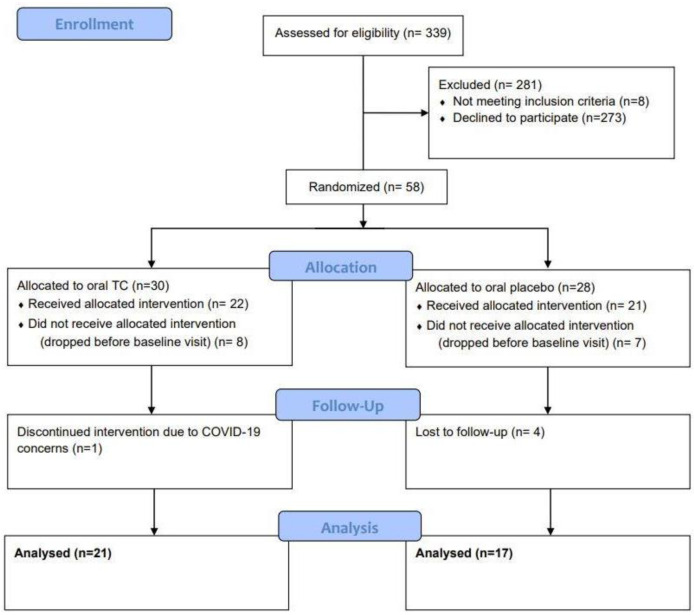
CONSORT Diagram.

Overall, 38 subjects completed the study with 21 subjects in the oral TC group (average age of 45 years old ranging from 25 to 65 years of age) and 17 subjects in the oral placebo group (average age of 44 years old ranging from 26 to 60 years of age). (Figure 2). Side effects were assessed throughout the study where patients were asked to report any adverse effects, changes in their gut related symptoms, or any hypersensitivity reactions like urticaria or new skin rashes.

### 2.3. Inclusion and Exclusion Criteria

Healthy females aged 25–55 who were overweight (BMI 25–35) were screened and assessed for study eligibility and those that had a Hemoglobin A1C that was between 5.5 to 7 were included. Subjects who had a known allergy to Triphala or *Terminalia chebula* were excluded. Subjects who had a history of malignancy, kidney disease, or chronic steroid use were excluded. Subjects who had chewed or smoked tobacco or vaped nicotine within the year prior to participation or who had a 5 pack-year history of tobacco were excluded. Subjects who had a history of anorexia or who had a history of taking serotonin-related supplements or systemic serotonin reuptake inhibitors were excluded. Subjects were not allowed to have any intake of pomegranate, walnut, or strawberry containing drinks or foods if they could not washout for two weeks prior to participation. Any subjects who were unable to discontinue topical medications from the treatment areas for two weeks or anyone that had any medical or cosmetic procedures to the face within 6 months of participation did not qualify. Systemic antibiotics or oral probiotics were not allowed for one month prior to participation and throughout the duration of the study. Postmenopausal or perimenopausal women or women who were pregnant or breastfeeding did not qualify.

### 2.4. Facial Imaging, Measurements of the Biophysical Properties of the Skin

All measurements were collected after subjects had adjusted to ambient conditions for 15 min in a climate-controlled room. The following measurements were collected at the initial baseline visit and at follow up visits.

The facial appearance of wrinkles was assessed using high-resolution facial photographs captured and analyzed by the BTBP 3D Clarity Pro^®^ Facial Modeling and Analysis System (Brigh-Tex BioPhotonics, San Jose, CA, USA). The “average severity” of wrinkles was calculated by measuring the depth and width of the wrinkles; this method has been previous validated [26].

Facial sebum production was measured with a Sebumeter^®^ SM 815 (Courage and Khazaka, Cologne, Germany). The SkinColorCatch^®^ (Delfin Technologies Ltd., Kuopio, Finland) was used to measure the facial erythema index of the skin.

### 2.5. Statistical Analysis

All parametric data results are presented as the mean ± standard error of the mean. Significance was assessed with the use of a Student’s *t*-test to assess the within-group (two tailed, paired) and between group (two tailed, unpaired) differences. A Bonferroni correction was applied for repeated measures. Any values of (*p* < 0.05) were considered statistically significant. Each subject served as their own control with within-group comparisons, as values reported at four and eight weeks were compared to baseline values, or 1.

## 3. Results

### 3.1. Sebum Excretion Rate

**Figure 3:**
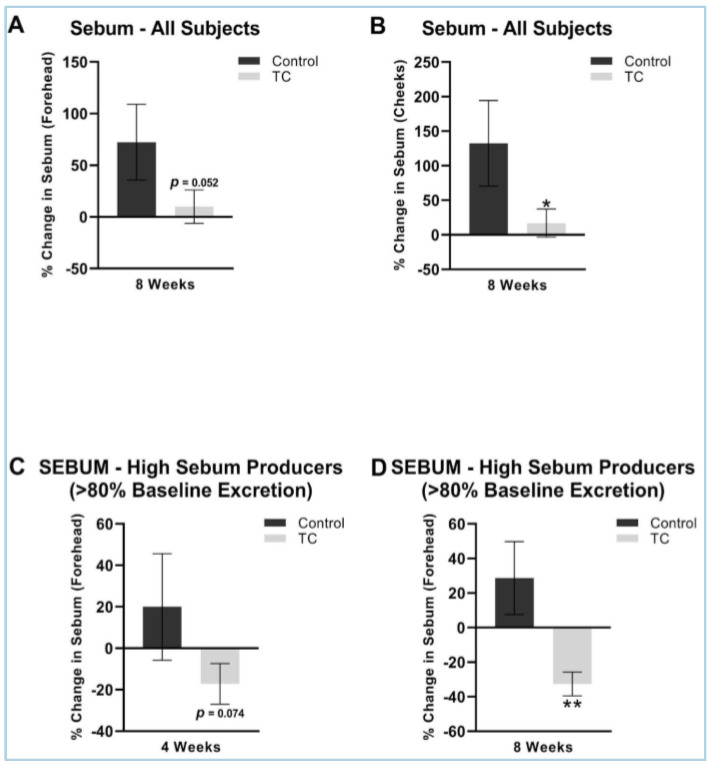
(**A**) Forehead sebum production after 8-weeks, all subjects. (**B**) Cheek sebum production after 8-weeks, all subjects. (**C**) Forehead sebum production after 4 weeks, high sebum producers (>80% baseline excretion). (**D**) Forehead sebum production after 8 weeks, high sebum producers (>80% baseline excretion). All presented data is compared to pretreatment baseline. N = 38 for (**A**,**B**), N = 15 for (**C**,**D**). * *p* < 0.05, ** *p* < 0.01.

The TC treatment had a lower increase in sebum at eight weeks compared to placebo on both the forehead (9.9% vs. 73% increase, *p* = 0.05) and on the cheeks (17% vs. 132% increase, *p* < 0.05). We performed a sub-analysis in those that were higher sebum excretors at baseline, as previous studies have shown that a sebum excretion rate of 80 ug/cm^2^ or greater correlates with more oily skin that is associated with the presence of acne [27]. The sub-analysis showed that TC supplementation had a significant decrease in the forehead sebum excretion rate compared to the placebo at four weeks (−17 decrease vs. 20% increase, *p* = 0.074) and at eight weeks (−33% decrease vs. 29% increase, *p* < 0.05). The results are shown in Figure 3.

### 3.2. Facial Erythema

**Figure 4:**
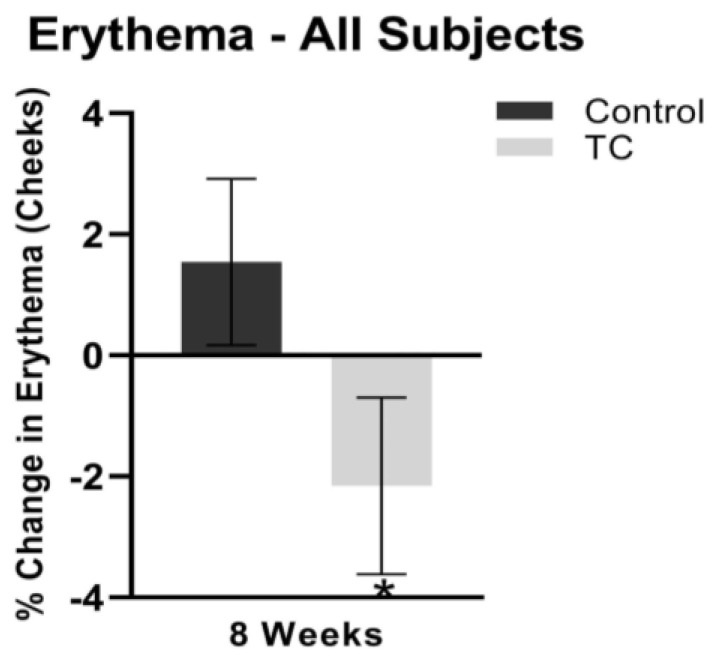
Cheek erythema after 8 weeks, compared to pretreatment baseline (all subjects). N = 38, * *p* < 0.05.

Cheek erythema decreased by 1.2% at four weeks, while the placebo treatment increased cheek erythema by 1.1% (*p* = 0.1). At eight weeks, cheek erythema decreased by 2.2% while the placebo treatment increased cheek erythema by 1.5% (*p* < 0.05). Results are shown in Figure 4.

### 3.3. Facial Wrinkles

**Figure 5:**
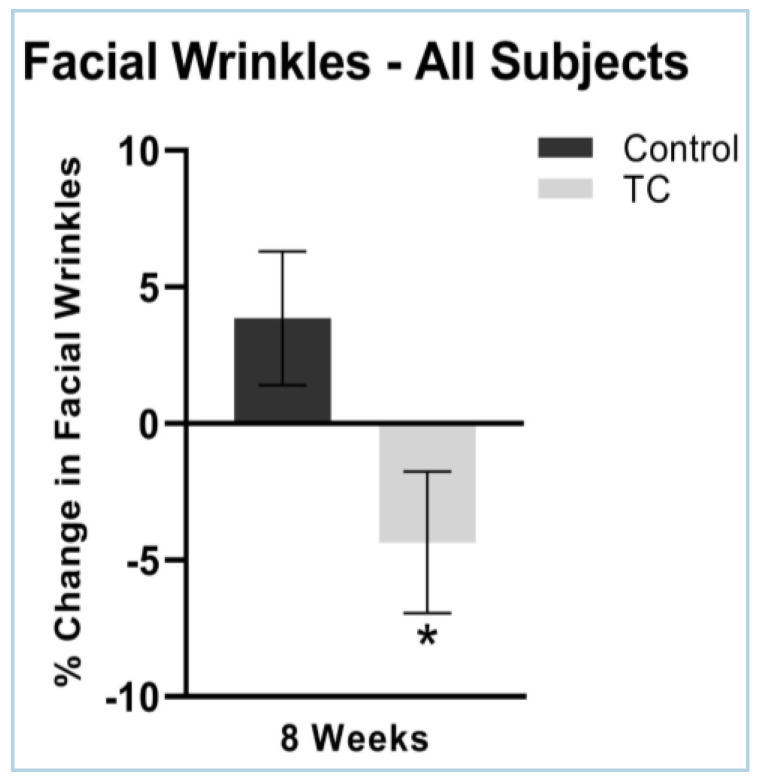
Facial wrinkles after 8 weeks, compared to pretreatment baseline (all subjects). N = 38, * *p* < 0.05. There were no side effects noted.

Facial wrinkles severity decreased by 0.34% at four weeks, while the placebo treatment increased wrinkle severity by 3.2% (*p* = 0.07). Facial wrinkles decreased by 4.4% in the TC group and increased by 3.9% in the placebo group after eight weeks of supplementation (*p* < 0.05). N = 38. Results are shown in Figure 5.

## 4. Discussion

Our work reveals that ingestion of 250 mg capsules twice daily for eight weeks of standardized TC extract reduces sebum excretion, especially in those that have higher baseline sebum excretion. Our results agree with previous clinical studies that have utilized herbal formulations where TC was one of the components [19,20]. However, in this study, TC was used as the sole standardized extract rather than as one of several components, which better isolates the effects of TC.

The decrease in sebum, especially among those that have higher sebum excretion, has important implications. The 33% reduction in those with high sebum excretion in the TC group is similar to the effect of oral spironolactone, which reduces sebum excretion by approximately 25–50% [28]. This warrants further study in populations that have higher sebum excretion such as those with acne or with seborrheic dermatitis.

Regarding skin aging, TC is reported to inhibit enzymes such as MMPs (MMP-1, MMP-2, MMP-3, MMP-9, MMP-12(elastase)), and hyaluronidase, while bolstering collagen expression/protein (COL1A1, COL1A2, COL1, COL3) and proteoglycans (PRELP, OGN), and it inhibits telomere shortening to improve replicative lifespan [29]. The effects of TC were evaluated on the localization and abundance of filaggrin (FLG), aquaporin, (AQP9) and loricrin (LOR) in full-thickness human skin biopsies [17]. Also, TC is reported to have antioxidant and anti-inflammatory effects [29] that can attenuate UVB damage and reverses pollution-induced skin damage [3,29]. This may be reflected in the improvement in the wrinkle severity and erythema measures in the TC group compared to the placebo group. While there was a decrease in cheek erythema in the TC group, the magnitude of the changes was small and warrants a longer study. The wrinkle severity decreased by 4.4%, which is similar to a previous study that evaluated the effect of oral supplementation with a standardized pomegranate extract [13].

A few interventional studies have investigated the presence of urolithin metabolites after intake of dietary fruits (strawberry and raspberry) and nuts (walnuts) in various study populations [30,31]. Investigators have also examined the association between different urolithin plasma profiles and health benefits, such as lowering cardiovascular risk in overweight populations [32,33,34]. TC extracts also contain ellagitannins, which are likely to be converted to urolithins by the gut microbiome. Interestingly, TC contains modified ellagitannin based on chebulic acid, but no work has been done to validate this assumption. Future assessments for the results seen here would be to assess for the carriage of the family *Eggerthellaceae* in the gut as previous studies have shown more profound effects when *Eggerthellaceae* is present in the gut [13].

This study has several limitations. The food restrictions were stringent in this study and allowed us to reduce the number of confounders and isolate the effect of TC. However, this may not be practical in a real-world setting. The study was done only on women, and the results may not be generalizable unless another study was performed on men. However, the focus on women allowed control for hormonal differences between men and women. Finally, this study focused on overweight women with BMIs between 25 and 35. Therefore, the results may not pertain to those that are outside of this BMI range.

## 5. Conclusions

Overall, our findings suggest that oral supplementation with standardized TC extract with highly enriched hydrolyzable tannins taken as 250 mg capsules twice daily for eight weeks may reduce sebum excretion, especially in those with high baseline sebum excretion (oily skin) and may improve facial appearance parameters like erythema and facial wrinkle severity. Our results warrant further study to better understand the mechanisms and the influence of the gut microbiome. Future studies should evaluate TC extract supplementation for acne, seborrheic dermatitis, and photoaging.

## Data Availability

The data is not publicly available. The data presented in this study are available on request from the corresponding author.
